# Acceptability and feasibility of a virtual community of practice to primary care professionals regarding patient empowerment: a qualitative pilot study

**DOI:** 10.1186/s12913-019-4185-z

**Published:** 2019-06-20

**Authors:** Carlos Jesús Bermejo-Caja, Débora Koatz, Carola Orrego, Lilisbeth Perestelo-Pérez, Ana Isabel González-González, Marta Ballester, Valeria Pacheco-Huergo, Yolanda del Rey-Granado, Marcos Muñoz-Balsa, Ana Belén Ramírez-Puerta, Yolanda Canellas-Criado, Francisco Javier Pérez-Rivas, Ana Toledo-Chávarri, Mercedes Martínez-Marcos, e-MPODERA group, Concepción Alejo-Díaz-Zorita, Cynthia A. Barbero-Macías, Fernando Borrell-Punzón, Beatriz Bueno-Rodriguez, Begoña Colmena-Martin, Isabel del Valle-de Joz, Juan Carlos Gamboa-Puñal, Concepción García-Valverde, Luis Miguel Gómez-Garzón, Arturo Gómez-López, Alejandro Hernaz-Guijo, Walter Nery Herrera-León, Irene Iniesta-González, Margarita Leza-Leza, María Aranzazu Melchor-Canelo, Sagrario Muñoz-Quirós-Aliaga, Ángela Oria-Fernández, Nuria Pertierra-Galindo, Mª. Dolores Prieto-Barbosa, Milagros Robledo-Vázquez, Marta Ruiz-López, M. Carmen Sánchez-Cruz, Mª. Ángeles Sánchez-de-Eusebio, Mª. Jose Seijas-Martínez-Echevarría, Carmen Paola Tovar-García, Jose Ignacio Vicente-Diez, Cristina Villanueva-Sanz

**Affiliations:** 1 Unidad de Apoyo Técnico, Gerencia Asistencial de Atención Primaria, Servicio Madrileño de Salud, Calle San Martín de Porres 6, 28035 Madrid, Spain; 2 Instituto Avedis Donabedian, Universidad Autónoma Barcelona, Calle ProvenÇa 293 pral, 08037 Barcelona, Spain; 3 Red de Investigación en Servicios de Salud en Enfermedades Crónicas (REDISSEC) ISCIII, Madrid, Spain; 4 Servicio de Evaluación y Planificación, Dirección del Servicio Canario de la Salud, Centro de Salud El Chorrillo, Camino Candelaria s/n, 38109, El Rosario, Santa Cruz de Tenerife, Spain; 5 Institut für Allegemeinmedizin. Johann Wolfgang Goethe-Universität, Theodor-Stern-Kai 7, 60590 Frankfurt am Main, Germany; 6 Centro de Salud Vicente Muzas, Gerencia Asistencial de Atención Primaria, Servicio Madrileño de Salud, Calle Vicente Muzas 8, 28043 Madrid, Spain; 7 EAP Turó, Vilapicina 8C, Barcelona, Spain; 8 Centro de Salud Monóvar, Gerencia Asistencial de Atención Primaria, Servicio Madrileño de Salud, Calle Monóvar 11, 28033 Madrid, Spain; 9 Departamento de Enfermería, Facultad de Enfermería y Podología, Universidad Complutense de Madrid, Plaza Ramón y Cajal, s/n, 28040 Madrid, Spain; 10 Fundación Canaria de Investigación Sanitaria, Centro de Salud El Chorrillo, Camino Candelaria s/n, 38109, El Rosario, Santa Cruz de Tenerife, Spain; 11 Departamento de Enfermería, Universidad Autónoma de Madrid, Calle Arzobispo Morcillo 4, 28029 Madrid, Spain

**Keywords:** Empowerment, Primary healthcare, Virtual system, Healthcare professional attitudes, Pilot study

## Abstract

**Background:**

Virtual communities of practice (vCoPs) facilitate online learning via the exchange of experiences and knowledge between interested participants. Compared to other communities, vCoPs need to overcome technological structures and specific barriers. Our objective was to pilot the acceptability and feasibility of a vCoP aimed at improving the attitudes of primary care professionals to the empowerment of patients with chronic conditions.

**Methods:**

We used a qualitative approach based on 2 focus groups: one composed of 6 general practitioners and the other of 6 practice nurses. Discussion guidelines on the topics to be investigated were provided to the moderator. Sessions were audio-recorded and transcribed verbatim. Thematic analysis was performed using the ATLAS-ti software.

**Results:**

The available operating systems and browsers and the lack of suitable spaces and time were reported as the main difficulties with the vCoP. The vCoP was perceived to be a flexible learning mode that provided up-to-date resources applicable to routine practice and offered a space for the exchange of experiences and approaches.

**Conclusions:**

The results from this pilot study show that the vCoP was considered useful for learning how to empower patients. However, while vCoPs have the potential to facilitate learning and as shown create professional awareness regarding patient empowerment, attention needs to be paid to technological and access issues and the time demands on professionals. We collected relevant inputs to improve the features, content and educational methods to be included in further vCoP implementation.

**Trial registration:**

ClinicalTrials.gov, NCT02757781. Registered on 25 April 2016.

**Electronic supplementary material:**

The online version of this article (10.1186/s12913-019-4185-z) contains supplementary material, which is available to authorized users.

## Background

Knowledge is key to the advancement of organizations. Learning and the implementation of practices, however, does not only rely on explicitly developed knowledge. Discussion, interaction, observation and the exchange of experiences between professionals all aid understanding of formal knowledge, which thus acquires meaning in line with proposals regarding social theories of learning [1], and imply an interaction between socially defined competences and personal experiences [2]. The community of practice (CoP) is one strategy that facilitates this type of learning [3].

A CoP, coined as a term in the 1990s [4], is, according to Wenger [5] “not just a website, a database, or a collection of best practices. It is a group of people who interact, learn together, build relationships, and in the process develop a sense of belonging and mutual commitment”. Having others who share their overall view of the domain and yet bring their individual perspectives on any given problem creates a social learning system that goes beyond the sum of its parts. A CoP reflects a common field (depending on the competences of the group) delimiting what is of interest to the group, the existence of a structure that encourages interaction and relationships among members, shared practices and a repertoire of resources such as ideas, experiences and information. A CoP differs from other types of groups in that support takes the form of formal and informal interactions between members and that the emphasis is on voluntary participation, shared learning and knowledge and the fostering of a sense of belonging [5]. While CoPs have largely developed in the business and education areas, they are increasingly being used in the health sector [6–10].

Information communication technologies have led to great growth in virtual CoP (vCoPs) in recent years, given their greater flexibility, ease and speed of communication compared to other communities [11]. However, their establishment involves more than merely importing the content of traditional communities into a new framework. Technological structures need to be created and barriers not present in face-to-face communities need to be overcome, mainly related to the fact that members may be many, located in different geographical areas, not know each other and can have different organizational cultures. Further barriers are related to the availability and use of the new technologies [12, 13].

Many of these limitations can be overcome by using discussion forums and video-conferencing for real-time exchanges and by appointing a facilitator to promote a greater sense of commitment and presence of participants and to manage content and resources [14, 15]. Online learning benefits from this type of communities of primary care professionals, related to a better management of their time constraints and to possible geographic barriers, promoting networking and collaboration. Online learning has also shown beneficial results in changing attitudes, increasing knowledge and skills, as well as possible benefits in patients’ related outcomes [16].

A recent review [13] found that vCoPs allow professionals to be attracted to interprofessional learning activities and collaboration among them that break down professional silos and barriers related to isolation, establishing a risk-free environment for participants that increases their participation and involvement. vCoPs allow the possibility for professionals to be involved in activities that promote interprofessionalism, an important aspect for the provision of quality care and the goal of accomplishing better health outcomes.

We developed a vCoP named e-MPODERA, a pun combining the terms “empower” and “e-learning techniques”, aimed at improving primary care professionals’ attitudes to the empowerment of patients with chronic conditions.

e-MPODERA vCoP was created as a gamified virtual knowledge-sharing CoP based on a Web 2.0 platform that offers learning activities and materials regarding patient’s empowerment. This platform uses forums as a main tool aimed at sharing experiences, practices and resources, and makes collaborative learning a powerful way for sharing knowledge, sensitize for empowerment concepts and develop real solutions to specific problems using open and inclusive technologies. The activities were designed using a competence framework based on 4 learning objectives and 12 core competences (Table 1). Gamification is based on ranking, points, goals and badges, added to interactive contents, like games, videos, and other individual and collaborative tasks. Content was developed considering patients’ health literacy and self-efficacy, self-monitoring, shared decision-making, coping capacities, etc. Some of the main subjects tackled were: attitudes towards empowerment, dimensions of empowerment, health literacy, share decision making, self-management, communication skills, special morbidity cases (i.e. obesity, diabetes mellitus, ischemic heart disease, lung disease, elderly patient, falls prevention, stroke, dementia), care of the caregiver, etc.

**Table 1:** Framework of learning objectives and competences

Learning Objective A: To improve awareness of patients’ and carers’ empowerment	
A1: To know the impact of patient empowerment and disempowerment	
A2: To know interventions and their effectiveness to improve patient empowerment	
Learning Objective B: To improve familiarity with the concepts of empowerment	
B1: To identify the dimensions of patient empowerment and skills related for applying them	
B2: To know the taxonomy related to patient empowerment	
B3: To know characteristics and theoretical and conceptual approach of initiatives that work on patient empowerment	
B4: To know new models and paradigms of patient-health professional relationships	
Learning Objective C: To improve perceived self-efficacy and confidence in one’s abilities	
C1: To apply strategies and tools to facilitate patient’s empowerment in daily practice	
C2: To identify barriers to empowerment and establish improvement actions	
C3: To develop content and materials on empowerment and / or feedback and contribute to the vCoP with new knowledge	
Learning Objective D: To improve credibility and expectations about favorable results of empowerment	
D1: To learn about Spanish, European and international initiatives of leaders or prominent organizations regarding empowerment	
D2: To enhance feedback and dialogue with professionals about the empowerment strategies used	
D3. To resolve myths and misconceptions about empowerment	

The objective of the present study was to pilot the acceptability and feasibility of the e-MPODERA vCoP before testing its effectiveness to improve primary care professionals’ attitudes to the empowerment of patients with chronic conditions in a cluster randomized control trial [17].

## Methods

### Study design

We used thematic analysis to perform this descriptive qualitative study [18]. Data was collected using the focus group technique because interactions between the participants enable them to share and reflect on their experiences [19]. The study was designed in accordance with the Consolidated Criteria for Reporting Qualitative Research (COREQ), a 32-item checklist for interviews and focus groups [20] (Additional file 1).

### Setting

The study was carried out in 3 urban general practices in the Madrid region attended to by 38 General Practitioners (GPs) and 35 Practice Nurses (PNs), with an assigned population of 63,298 people.

### Participants

We purposively sampled for the focus group discussions 12 professionals, 6 GPs and 6 PNs, out of the 26 professionals that participated in the e-MPODERA vCoP, considering not only their level of participation in the vCoP (high, medium and low), based on the degree of participation in forums, comments and entries on the platform, but also age, sex and experience in primary care (Table 2).

**Table 2:** Socio-demographic characteristics of the participants in the focus groups

ID number	Focus group	Age	Sex	Primary care experience (years)	vCoP participation level^a^
GP 01	1	53	M	25	Medium
GP 02	1	46	M	17	Low
GP 03	1	51	F	23	High
GP 04	1	58	F	34	Medium
GP 05	1	62	F	27	Low
GP 06	1	50	F	17	Low
PN 01	2	42	F	19	Low
PN 02	2	47	F	19	Low
PN 03	2	42	F	17	Low
PN 04	2	60	F	10	High
PN 05	2	29	M	3	Medium
PN 06	2	60	F	28	Low

GP: general practitioner (*n* = 6); PN: practice nurse (n = 6)

^a^The level of participation was defined considering the degree of participation in forums, commentaries and entries in the platform

During the piloting, content was gradually incorporated into the vCoP over a 4-month test period (June to September 2016). All participants entered the vCoP and 31% contributed with comments. The topics inviting most comments were health literacy, shared decision-making and communication.

### Procedure

Two focus group sessions were held, one with 6 GPs and the other with 6 PNs, in November 2016. The focus group sessions lasted between 60 and 90 min, were held in a neutral venue and were facilitated by a moderator (CJBC), who led the discussion, and 2 assistants (YR, MMB), who took notes and audio-recorded the sessions. The moderator and assistants were qualitative researchers with training and experience in the field and with no direct relationship with the participants. Each group session commenced with a description of the objectives and a request for consent to both participation and audio-recording. After each group session, the moderator and assistants discussed the sessions and also took notes at these meetings.

We prepared guidelines for the focus group which contained the topics to be explored (Table 3), although the moderator was also allowed to inquire further regarding issues that emerged during the sessions.

**Table 3:** Focus group guidelines (topics)

•Problems that have hindered participation in the vCoP •Reasons for participating or not participating in the vCoP •vCoP barriers to improving patient empowerment •How could the vCoP and e-MPODERA bring about change in professional practice that promoted patient empowerment? •What resources would foster this change? •How do you think this kind of vCoP improve the exchange of knowledge? •What activities would you like the vCoP to offer? •What activities would you like to eliminate from the vCoP? •The role of the vCoP facilitator	

### Data analysis

The interviews were audio-recorded and later transcribed verbatim. Two researchers (CJBC, MMM) first read the transcriptions for both groups in their totality to ensure familiarity with the texts. Using a thematic analysis approach, the transcriptions were then coded independently by the 2 researchers (CJBC, MMM) using the ATLAS.ti software program, with open coding followed by categorization and abstraction.

Following the steps identified by Braun & Clarke [21], we followed six simple steps to conduct a thematic analysis: 1) familiarizing ourselves with the data; 2) generating initial codes; 3) searching for themes; 4) reviewing the themes; 5) defining and naming the themes; 6) preparing the report.

Researchers (CJBC, MMM) met regularly to discuss and debate findings until consensus was reached [22, 23]. To enhance validity, findings were reviewed by other members of the research team who contributed to the thematic analysis. The findings were transmitted to the focus group participants to obtain their feedback [19].

### Ethics

The study complied with Good Clinical Practice standards and the principles of the latest version of the Helsinki Declaration (Brazil, 2013) and was approved by the Clinical Research Ethics Committee of Ramón y Cajal University Hospital in Madrid (ACTA 303), Nuestra Señora de Candelaria University Hospital in Santa Cruz de Tenerife (PI-18/16) and IDIAP Jordi Gol in Barcelona (C2748). Confidentiality and anonymity were guaranteed in accordance with Spanish Law 15/1999 on personal data protection and all participants in the study gave their informed consent.

## Results

The participants expressed great interest in the vCoP learning environment, which allowed them to access content even from home and at times that suited them always using a personal computer. They highlighted the usefulness of vCoP material like tutorials, videos and demonstrations, indicating that this content led them to reflect on how they approached clinical practice; they also reported that the interaction with other professionals led them to contemplate other approaches to address the empowerment of patients with chronic conditions. However, they pointed some difficulties in accessing the vCoP.

See Table 4 for more detailed information on the emerging themes and subthemes.

**Table 4:** Emerging themes and subthemes

THEME	THEME	THEME
Novel kind of training	Benefits for patient empowerment	Difficulties using vCoP
**SUBTHEMES**	**SUBTHEMES**	**SUBTHEMES**
Flexible accessibility for learning Provision of useful materials in consultation Information based on evidence Possible ways to address problems Motivation for learning	Opportunity to reflect on their own clinical practice Share tips and demonstrations. Interaction / exchange of experiences among professionals Resources to motivate the chronic patient	Technological difficulties Tutorials that do not facilitate the use of the platform Information in non-native language No availability of time to access the work environment Little ease for access from home

### vCoP: a novel kind of training

Clinical practice is perceived by the participants as taking place in a context of multiple demands - by the organization, the population and society as a whole. The participants referred to the need to have new and up-to-date resources, easily implemented as a work aid and capable of responding effectively to the needs of patients with chronic conditions while adapting to the context of their health centre.

The vCoP was viewed as a learning mode that met participants needs through activities that could be accessed in a flexible way according to the time available, including from home.

*“And it's practical, they give you a day a week when, if you want, you can see the class live and it's a video-conference and if you download anything it’s recorded and you watch it when you can, the hour and a half or two hours that the class lasts and then you see the instructor, who explains things and you ask. I find that very useful*.” PN 04

This form of learning was considered of interest insofar as it could provide the participants with useful materials, defined as up-to-date resources allowing easy access from the point of consultation and responding to the needs of patients. GPs perceived a greater need for this type of information accessible at the point of care, information not available to them from other available resources.

*“Specific things that help us, that facilitate things with the patient because we do not have time ...and when you don’t have time, of course, you can’t be there, looking through a hundred thousand things to see which is the most interesting.”* GP 05

In relation to the potential of the vCoP as a training element, the professionals indicated that it should contain up-to-date, specifically primary care work-related and evidence-based information previously selected as especially relevant by those responsible for development of the vCoP. This information should also be easily accessible from the point of consultation in order to ensure more rapid answers than provided by existing resources.

*“If you had that information directly, scientific of course, in 5 minutes you could read it or a summary paragraph. Many of the platforms do not address that and if we have to find things ourselves, we have to do very exhaustive searching that takes a long time.”* GP 02

Regarding the opportunities offered by the vCoP for interaction and the exchange of experiences, mainly through forums and interactive video-conferencing sessions, participants indicated that this helped them consider alternative approaches to similar problems or clinical situations applicable to routine practice. Both groups of professionals considered useful opportunities for interaction and exchange of experiences with other professionals.

*“I found them very interesting because I found them useful for practice ... with the patients, to deal with some issues that maybe hadn’t occurred to me, that I’d looked at superficially, to notice and consider them.”* GP 01

For the vCoP to become consolidated as a training tool, it requires the appointment of an individual or team to select relevant and easily accessible information from the large amount of information available and to assume a motivational role in encouraging professionals to enter the community, consult information, participate in forums, etc. … figure that both PNs and GPs considered necessary.

*“Facilitators. People who filter maybe and who help us a little with managing things at a specific moment. The most important things. So that you don’t have to, what she said before, access a load of information, that there’s a kind of filter of what’s most important or most relevant.”* GP 06

### vCoP: benefits for patient empowerment

The vCoP activities were considered, by both groups of professionals, to be an opportunity to examine and reflect on approaches to chronic patients in their clinical practice and to analyse alternative approaches. Tutorials and demonstration videos were considered especially useful as they enabled patients to be managed from a more comprehensive perspective with the goal of empowering them.

*“... I was doing activities, many that I found very appropriate and good for teaching. The truth is, they even make you think a little and elaborate on things. It made you discuss sometimes … what you usually do in consultations.”* GP 01

The nurses placed more emphasis on the usefulness of the vCoP in terms of tips and demonstrations that could be used with their patients to address specific issues

*“That's what I was referring to, like when someone helps you see the light, so [ you tell the patient] ‘protect the fingertips’ or ‘take some holidays’, those phrases that seem silly when it comes to consulting, I used them.”* PN 02

New approaches and ways of doing things, as one participant commented, could be learned from the opinions, judgements, experiences and procedures of other professionals. The interaction between professionals fostered by the vCoP was considered to be an opportunity to advance learning.

*“... you access to see more things, to see what they comment, the impressions of another person, the game that’s played between colleagues, what one does, what the other does, we could learn new ways of acting.”* GP 01

Some expectations were not fulfilled by the vCoP in this pilot phase, specifically by the PN group. These include the availability, to use during consultations, of previously prepared resources aimed specifically at reinforcing patient knowledge. Among these resources, special mention was made of information resources for patients or to work conjointly with them.

*“Yes, maybe a library of resources with teaching materials to help the patient would be useful, which we, in the consultation … well, if they are young or are used to the internet, we could easily download useful information on their chronic illnesses and give them that didactic material to train them too.*” PN 05

Since one frustration for professionals was patients not adhering to guidelines or advice, a suggestion was to include resources aimed at stimulating the interest of patients in addressing adherence, as both GPs and PNs manifested.

*“As a professional, maybe I miss some kind of methodology to motivate them, some line or strategy, that’s to say, techniques or something, you know? To emphasize things so they leave the consultation motivated to say, you're right, you know what I mean?”* PN 02

### vCoP: difficulties using it

During the focus group sessions, the study participants pointed to several difficulties with using the e-MPODERA vCoP, with the main difficulty generally related to access issues.

Participants referred to great difficulty in using vCoP in their workplace, since the computers there had operating systems and browsers that made it difficult to access the platform. This technical difficulty was due to equipment obsolescence, not to participant capacity to use the technology, and there were no differences in this respect between the GPs and PNs. When software problems, such as inexplicable system crashes and error messages, occurred during workplace use, the professionals became frustrated and stopped using the tool.

*“If at work in the centre it’s impossible, we don’t have access, access is impossible. You click, it blocks, the page stays blank, you go back and it’s just impossible.”* PN 04

Not all work centres had the same difficulties, as some centres with up-to-date computer equipment offered better accessibility.

Another access difficulty for participants was to find a suitable space and time to use the vCoP in a meaningful way. Both GPs and PNs reported how the burden of patient care and administrative tasks consumed their working hours, leaving very few opportunities for innovation and learning. Consequently, many participants reported accessing the vCoP from home, although here too fitting access in with family demands was not always easy.

*“Then the times that I’ve been able to connect have always been at home, which just seemed a bit … uff. Because then from home, it depends on things, because you have more or less time.”* PN 05

Other difficulties described by the participants referred to access to the virtual platform itself. It was always necessary for the user to remember their user name and password information, and options to recuperate this information were less than satisfactory. In general, PNs expressed more difficulties related to limitations on access to the platform.

*“For me in practice access has sometimes been difficult. The first part, doing the survey went very well, that was easy. Then you go back and what’s the password. I took a photo and even with a photo it was difficult to access.”* PN 02

The tutorial designed to facilitate use of the vCoP was not considered useful, given its location in the platform and the fact that it was considered complicated.

*“Well, other than that, the tutorial was a bit complicated. We talked to one of them [on the platform] and I think it was located in ... or in another place, because it was at the side, right? The truth is I did not understand very well at the beginning, when I got into the tutorial.”* GP 03

While most of the documents and activities for use by the professionals were in Spanish, some were in English. Because the professionals, specially the PN group, had to use online translators, these documents were less consulted and less likely to be read and used.

*“I would ask just one thing, it’s probably also my ignorance. That not everything would be, that not much information would be in English.”* PN 02

## Discussion

Professionals generally viewed virtual learning environments like e-MPODERA vCoP with interest. Use of the Internet for ongoing training purposes has increased in recent years. In the health area, a significant percentage of health professionals use the Internet daily for work-related reasons, mainly to locate information for patients, but also for self-managed ongoing training via online reading and learning activities [24].

Participants in the study considered important to have up-to-date equipment and resources that responded to the needs posed by patients in consultations, and most especially, information that was not available from other resources, as the goal is to be able to provide rapid responses. Participants preferred to have resources that they could use in routine practice, such as demonstration videos and educational materials, but available in the local language. Barnett [25] confirms that perceived usefulness is a key predictive factor in any intention to use a vCoP.

The vCoP was perceived as an encyclopaedia that would always be available for use when necessary [26]. Barnett [25] reported that the resources most valued by doctors-in-training and tutors are the possibility of sharing clinical practice guidelines and other documents, followed by discussion forums. The sustainability and maturation of a vCoP relies on resources such as technical documents, demonstration materials, videos, photos, etc. available in repositories or in information sections in external websites [10, 27–29]. Another advantage of the vCoP pointed out by participants was that general or specific information was available almost immediately [30]. As McLoughlin et al. [13] outlined, this aspect is key for the engagement of professionals in the vCoP.

Participants also emphasized the speed of responses from the rest of their team [25, 28]. Indeed, interaction between the vCoP participants in general was considered to be crucial, as it offered the opportunity to exchange experience, to learn of alternative approaches to patients with chronic conditions, to examine, reflect on and analyse other perspectives related to routine practice and to observe the skills and even practical demonstrations of other participants. The most valued vCoP areas regarding the empowerment of chronic patients were the forums and interactive sessions such as video-conferences with external experts.

Interaction between vCoP participants is an aspect highlighted in other studies, not only in relation to exchanging documentation, but also in terms of resolving doubts and problems through the contributions of other participants sharing experiences and exchanges aimed at improving knowledge and skills [26, 29, 31]. Interaction between professionals in the same field of work in normal working conditions is crucial, and in a vCoP, the interaction format that generates the most shared knowledge is that which combines face-to-face and online interactions, followed by face-to-face interactions [25, 28, 31]. Barnett [29] reported that participants using a vCoP stated that they benefited mainly from online interactions aimed at generating trusting relationships, but also from the confidence generated by prior knowledge shared by participants.

Certain barriers to interaction were identified. Active participation by all members was considered by some participants to be crucial for the proper functioning of the vCoP [29]. Indeed, under-participation can lead to failure of this form of learning [13]. Even so, the less active participants were of the opinion that they also benefited from reading the contributions of the other participants, even though they themselves could not participate actively, for instance, due to a lack of time [29]. In some cases, non-participation was due to a fear that a contribution might be trivial or irrelevant [30]. However, in most vCoP 90% of participants act as observers and never contribute, 9% contribute just occasionally and 1% are always active [32]. As McLoughin et al. showed [13], several strategies have been reported to increase participation such as increasing trust among members, focusing vCoP on common interests or focusing vCoP on the patient by providing resources based on easily accessible evidence.

The vCoP facilitator played an important role, according to the participants in the study. A facilitator motivates members to participate and also helps by filtering more relevant and important information, especially that based on evidence. On the other hand, facilitators play an important role in ensuring clear rules, focusing the discussions and promoting the involvement and respect among the members [13]. In this study, the facilitator was a primary care professional who participated by leading and developing the vCoP. In different vCoPs analysed by Barnett [33] the facilitator may be the person who initiates and launches the vCoP or may emerge from the group of participants, especially from among more active participants. The role of facilitators is usually to enhance cooperation, but they also can ensure that the rules of engagement are clear, ensure focus on discussions, establish procedures for member inclusion and lead commitment to and maintenance of the vCoP. The facilitator also identifies relevant content and saves it correctly to bring it back when it is necessary [9]. In this study, clinically active facilitators were key to the development of the vCoP in terms of recognizing the contributions of participants and in guiding them [29, 33]. Another contribution of the facilitators was notifying new entries and frequent participants.

A vCoP needs a technological infrastructure that will support online operation and use and so requires more careful design than traditional learning formats. As pointed out by Barnett [33], ensuring that a technology is easy to use enhances the potential success of a vCoP. In our study we observed difficulties fundamentally related to access, such as obsolete or incompatible browsers and the lack of suitable additional software to access all the available resources. Another identified difficulty was the need to create and remember an access password. Such difficulties lead to frustration and abandonment of vCoPs.

Other difficulties identified by the professionals that participated in our study are common to the ones found in the literature [24, 31, 33–36] such as slow connections, problems downloading information and documents, workplace restrictions on Internet access by employees, incompatibilities with tablets and mobile phones and audio problems.

The main difficulty reported by the professionals in our study was a lack of time, which hinder access to learning environments, especially from the workplace [35]. The main advantage of a vCoP for professionals, according to previous studies [26, 31], is the flexibility of this kind of learning environment, as it allows access at the time and in the place considered most convenient. Most professionals in piloting of the e-MPODERA vCoP preferred to access the vCoP resources from home (43.9%), followed by those who accessed from both home and work (33.2%) [24]. Technical problems, as corroborated by several authors [24, 31, 37], combined with a lack of time at work led many participants to use the vCoP in the home setting, even though they would have preferred use in the workplace as most suitable from a training perspective.

One limitation of the study is that it was carried out in 3 urban, city-based, general practices (in Madrid) and so excluded rural centres. Therefore, it was not possible to access the perceptions of professionals working in more isolated settings. Besides, only a limited number of professionals were invited to participate in this pilot study (26 primary care professionals) and vCoP enrich with the participation of more professionals, not being able to reach a saturation of the speech. Another limitation was that the duration of use of the vCoP (6 months) was probably not long enough for relationships of trust between the participants and a feeling of belongingness in a community to develop. The difficulties accessing the platform did not help in this matter. Finally, perceptions of the vCoP may have been affected by the fact that participants in the study, while employed by the Madrid Health Service, work in different autonomously managed health centres; thus, exchanges between professionals may have been limited by a certain homogeneity in the organizational criteria of the different centres.

## Conclusions

The results from this pilot study show that the e-MPODERA vCoP was considered useful for learning how to empower patients. However, attention needs to be paid to technological issues, and the time demands on professionals. We collected relevant inputs to improve the features, content and educational methods to be included in further vCoP implementation (Table 5).

**Table 5:** Facilitators and barriers to the use of the vCoP

**Facilitators**	
The use of information and knowledge technologies allows the development of vCoP with flexible accessibility for the training of health professionals. The inclusion of evidence-based and updated materials is a necessary and useful tool for healthcare activity. Maintaining and providing useful and updated information motivates the use and functioning of the vCoP. The vCoP is a medium that allows interaction between professionals from different locations and enables sharing approaches, opinions, experiences and ways of performing. The presence of easy-to-understand tutorials, with the necessary information for the use of vCoP, would improve access to the included resources and participation.	
**Barriers**	
The use of out-of-date or inappropriate hardware and software limits and hinders access to and participation in a vCoP. The inclusion of documents and resources in non-native language causes difficulties in understanding information. The time restrictions in work environments can limit access to a vCoP.	

## Additional file

Additional file 1: COREQ (COnsolidated criteria for REporting Qualitative research) Checklist. (PDF 497 kb)

## Data Availability

Not applicable.

## Abbreviations

CoP: Community of Practice
e-MPODERA: Effectiveness of a Virtual Intervention to Improve Healthcare Professional Attitudes to Patient Empowerment
GP: General Practitioner
PN: Practice Nurse
vCoP: Virtual Community of Practice
